# Robust Magnetic Fingerprint Positioning in Complex Indoor Environments Using Res-T-LSTM

**DOI:** 10.3390/s25247464

**Published:** 2025-12-08

**Authors:** Kaihui Guo

**Affiliations:** School of Architecture, Soochow University, Suzhou 215008, China; 20204041002@stu.suda.edu.cn; Tel.: +86-188-6218-5709

**Keywords:** magnetic field, residual networks, transformer, LSTM, magnetic positioning

## Abstract

With the increasing demand for indoor location-based services, magnetic-fingerprint-based positioning has emerged as a promising complementary solution in scenarios lacking WiFi coverage. However, the dynamic nature of indoor environments, architectural complexity, and variations in pedestrian walking speeds can lead to stretching, compression, and distortion of magnetic fingerprint sequences, making it challenging for traditional sequence-matching algorithms to maintain stable positioning performance. To address these challenges, this paper proposes a magnetic-fingerprint-based positioning model that integrates residual networks (ResNet), transformer, and LSTM, referred to as Res-T-LSTM. Within the overall architecture, the ResNet module extracts deep local spatial features of magnetic fingerprints, and its residual connections effectively mitigate gradient attenuation during deep network training. The transformer module leverages self-attention mechanisms to model long-range dependencies and global contextual information, adaptively emphasizing key magnetic variations to enhance the discriminability of the feature representations. The LSTM module further captures the dynamic temporal evolution of magnetic sequences, improving robustness to variations in walking speed and sequence stretching or compression. Experimental results show that the proposed model achieves excellent performance across four smartphone-carrying postures, yielding an average positioning error of 0.21 m.

## 1. Introduction

In recent years, indoor positioning technology has advanced rapidly, becoming a major research focus with broad applications in the internet of things, intelligent transportation, smart healthcare, and industrial manufacturing. The widespread adoption of smartphones and integration of multi-sensor technologies—such as accelerometers, gyroscopes, magnetometers, WiFi, Bluetooth, and UWB—have significantly boosted location-based services, which demand high accuracy, real-time performance, and stability. In open outdoor environments, global navigation satellite systems, including GPS, BeiDou, GLONASS, and Galileo, provide stable, reliable, and high-precision positioning services [1]. However, indoor environments contain obstacles—such as walls, floors, and metallic structures—that significantly attenuate satellite signals and induce multipath effects, reducing visible satellites and degrading signal quality. Consequently, the applicability of traditional satellite navigation systems is severely limited indoors, significantly affecting positioning accuracy and reliability. Therefore, achieving high-precision, low-cost, and robust positioning in complex, signal-degraded indoor environments remains a critical challenge requiring urgent attention.

To address these challenges, extensive research has been conducted in both academia and industry on indoor positioning technologies in recent years [2,3]. One category of methods relies on wireless propagation models, which estimate pedestrian positions based on received signal strength [4,5,6]. Another category involves fingerprint-matching algorithms, which associate fingerprints with geometric spatial locations and estimate positions using the collected signal measurements. In addition, pedestrian dead reckoning (PDR) algorithms [7] leverage inertial sensors to achieve continuous localization through step detection and heading estimation. However, cumulative errors in inertial sensors inevitably cause long-term drift in the estimated trajectory, necessitating external technologies for correction [8].

Earth’s stable and unique geomagnetic field offers a reliable basis for developing and implementing magnetic fingerprinting technology [9]. In open outdoor environments, the geomagnetic field is relatively uniform, showing only minor variations. However, indoors, metals such as iron in reinforced concrete and copper in pipelines disturb the natural geomagnetic distribution, generating significant spatial variations. These variations provide a reliable basis for constructing location-specific and distinctive magnetic fingerprint databases. A triaxial magnetometer is typically employed to sample spatial geomagnetic features. Depending on the processing method, three types of magnetic fingerprint representations can be derived. The first approach employs the magnitude of the magnetic field vector (magnetic flux density) to construct a one-dimensional magnetic fingerprint [10]. This method is simple and noise-robust but discards directional information, potentially limiting its discriminative capability in certain scenarios. The second approach constructs a two-dimensional fingerprint by decomposing the magnetic field into vertical and horizontal components [11]. This approach retains directional information, enhancing fingerprint uniqueness. However, this decomposition typically relies on orientation data from a gyroscope. Low-cost inertial measurement units often exhibit low accuracy and high noise, leading to angle estimation errors. Such errors may propagate into the magnetic components, causing deviations and reducing matching accuracy. The third approach directly utilizes raw triaxial magnetic field data (X, Y, and Z components) to construct a three-dimensional magnetic fingerprint [12]. This method preserves the most comprehensive information and theoretically provides the highest discriminative capability. However, as the magnetometer is typically mounted on a mobile platform (e.g., smartphone or robot), its coordinate system continuously changes with the platform’s orientation. Consequently, identical spatial locations may produce inconsistent triaxial readings in the device’s coordinate frame. This issue significantly limits the practical application of 3D magnetic fingerprints, necessitating either attitude compensation mechanisms or orientation-invariant feature representations.

Deep learning has been widely applied in indoor positioning due to its powerful feature extraction capabilities, which enhance both accuracy and stability. Among deep learning models, convolutional neural network (CNN), recurrent neural network (RNN), and their variants are widely employed in indoor positioning for their effectiveness in processing sequential data and extracting spatial features. Imran Ashraf et al. proposed MINLOC, a CNN-based indoor localization system that exploits magnetic field patterns [13]. Mahdi Abid et al. developed a CNN-based indoor magnetic fingerprinting system augmented with an attention-GRU module [14]. To further improve positioning performance, RNNs and their variants, particularly LSTMs, have been applied in indoor localization. Xuyu Wang et al. introduced DeepML, a deep LSTM-based indoor localization system that integrates magnetic and ambient light data [15]. Despite the success of CNNs and RNNs, robust magnetic feature extraction remains challenging in complex and dynamic indoor environments, particularly in large-scale scenarios. Magnetic field distributions are affected by multiple factors, including building structures, metal objects, electronic equipment, and human activity. The combined influence of these factors renders magnetic features highly complex and difficult to model. Moreover, variations in magnetic fingerprint sequence lengths—resulting from differences in pedestrian walking speeds—and sensor heterogeneity over time further complicate feature extraction.

To address the limitations of existing deep learning models in capturing complex magnetic field features, this paper proposes a novel hybrid architecture, Res-T-LSTM, which integrates ResNet, transformer, and LSTM to achieve high-precision representation of magnetic fingerprint data. Specifically, ResNet extracts deep local spatial features of magnetic fingerprints, the transformer models long-range dependencies and global contextual information via self-attention mechanisms, and LSTM captures dynamic patterns in temporal sequences. This multi-module architecture fully leverages spatiotemporal features, enabling comprehensive characterization of complex magnetic field distributions and significantly enhancing indoor positioning accuracy. Compared with conventional single-network structures, Res-T-LSTM shows clear advantages in feature representation and positioning performance, representing a significant innovation in magnetic fingerprint-based localization. The main contributions are summarized as follows:

1. Hybrid Multi-Module Architecture Design: We propose a novel Res-T-LSTM architecture integrating ResNet, transformer, and LSTM, enabling unified extraction of local spatial features, modeling of global contextual dependencies, and capturing dynamic temporal patterns, thereby overcoming the limitations of single-network models in magnetic feature representation.
2. Comprehensive Exploitation of Spatiotemporal Magnetic Fingerprint Features: ResNet extracts deep local spatial features, the transformer models long-range dependencies and global context, and LSTM captures dynamic temporal patterns. This combination enables thorough characterization of complex magnetic field distributions, improving indoor positioning accuracy.
3. Significant Improvement in Positioning Performance: Compared with conventional single-network structures, Res-T-LSTM shows clear advantages in magnetic fingerprint feature representation and localization accuracy, validating the innovation and practical value of the hybrid architecture.

The paper is organized as follows: Section 2 reviews related research, establishing the theoretical foundation for the proposed method. Section 3 describes the core principles and implementation details of the proposed algorithm. Section 4 evaluates the algorithm’s performance in practical scenarios through experiments. Finally, Section 5 concludes with a summary and discussion.

## 2. Related Work

Indoor positioning technologies are diverse, with magnetic fingerprint-based methods attracting significant attention owing to their low cost and independence from additional hardware. These methods are broadly categorized into traditional fingerprint matching algorithms and machine learning-based approaches. Traditional fingerprint matching algorithms include several classical techniques. The K-nearest neighbors algorithm [12] determines the target location by identifying the nearest reference points in the feature space. The weighted matching algorithm [16] enhances matching accuracy by assigning different weights to features. The dynamic time warping algorithm [17] is effective for time-series data, aligning sequences of varying lengths by dynamically adjusting the time axis to accommodate environmental fluctuations. Additionally, the Euclidean distance method measures similarity by computing the straight-line distance between feature vectors. Machine learning-based algorithms have further advanced magnetic fingerprint localization. Support vector machines provide robust classification and regression by identifying optimal hyperplanes in high-dimensional spaces. Neural networks [18], leveraging their nonlinear modeling capacity, learn complex spatial patterns in magnetic fingerprint data, enabling more precise location predictions.

### 2.1. Traditional Magnetic Fingerprint Positioning

Magnetic fingerprint matching is a widely adopted indoor positioning approach that leverages ambient magnetic field features for accurate localization. Over the years, researchers have explored magnetic fingerprinting from multiple perspectives. Subbu et al. [19] employed magnetic field intensity as a one-dimensional fingerprint, establishing the foundation for subsequent studies. Li et al. [20] improved positioning accuracy by incorporating vertical and horizontal magnetic field components into a two-dimensional fingerprint. Le et al. [21,22] further advanced the field by adopting a three-dimensional magnetic field representation, significantly enhancing positioning precision. Despite these advances, magnetic fingerprinting faces several challenges. A key issue is fingerprint mismatching, often arising from the inherent ambiguity of magnetic signals. To address this, multi-sensor fusion has become a prominent research direction. Sun et al. [23] proposed a method integrating PDR with geomagnetic data via a genetic-particle filter, improving reliability. Huang et al. [24] introduced the MAIN system, which employs magnetometer arrays to capture spatial magnetic variations, substantially reducing horizontal positioning errors. Pan et al. [25] developed a WiFi/geomagnetic fusion method that filters noisy WiFi signals and integrates them with magnetic data, demonstrating strong performance in environments with sparse WiFi coverage. Wang et al. [26] further enhanced positioning accuracy by 33.2% using a method that fuses WiFi, PDR, and geomagnetic data via enhanced particle filtering and iterative magnetic matching. Nevertheless, multi-sensor fusion increases computational complexity and requires more capable hardware. It may also introduce sensor-related errors, compromising system stability. Concurrently, conventional fingerprint matching methods suffer intrinsic limitations, making it challenging to resolve magnetic signal ambiguity and further improve robustness and accuracy.

### 2.2. Machine Learning-Based Magnetic Fingerprint Positioning

Machine learning techniques have emerged as a key research focus and are widely applied in magnetic fingerprint positioning. Geomagnetic positioning systems often enhance accuracy and robustness by leveraging deep neural networks. Numerous studies have explored this area in depth. Lee et al. [27] proposed the AMID system, which employs a CNN to extract features from 32×32 magnetic sequence images and integrates a multi-layer perceptron to classify 99 landmarks accurately. Experiments demonstrated excellent positioning performance in environments such as corridors and atriums. Abid et al. [28] introduced a CNN-based system that analyzes recurrence plots of geomagnetic sequences, significantly outperforming traditional approaches and reducing mean distance error sevenfold. Wang et al. [29] developed the DarLoc system, combining a multi-branch CNN with a LSTM network to handle multi-scale sequences. DarLoc leverages feature enhancement techniques to improve positioning performance in complex environments. Lei et al. [30] introduced DeFLoc, a vehicle positioning system that reconstructs FM fingerprint maps using CNNs, reducing data collection requirements by 60% and improving efficiency in large-scale deployments. Fan et al. [31] proposed a 1D CNN with multi-feature fusion for magnetic anomaly detection. By integrating Hilbert-Huang and discrete wavelet transforms for feature extraction, it achieves high accuracy in detecting magnetic anomalies. Wang et al. [32] applied a multi-scale transformer with attention mechanisms to magnetic spatiotemporal positioning, providing new strategies to address spatiotemporal variations. Online magnetometer calibration further improves environmental and device consistency. Yan et al. [33] developed a fusion positioning system based on ResNet-GRU-LSTM, integrating geomagnetic and PDR data. This system exploits the complementary strengths of different modalities, improving positioning precision. Although these machine learning algorithms perform well in specific environments and have advanced indoor positioning capabilities, they still exhibit notable limitations. They are often sensitive to environmental changes, show limited generalization across diverse indoor spaces, and struggle to capture the complex spatiotemporal dependencies embedded in fingerprint sequences. These limitations restrict their robustness and scalability in real-world applications.

## 3. Methodology

**Figure 1:**
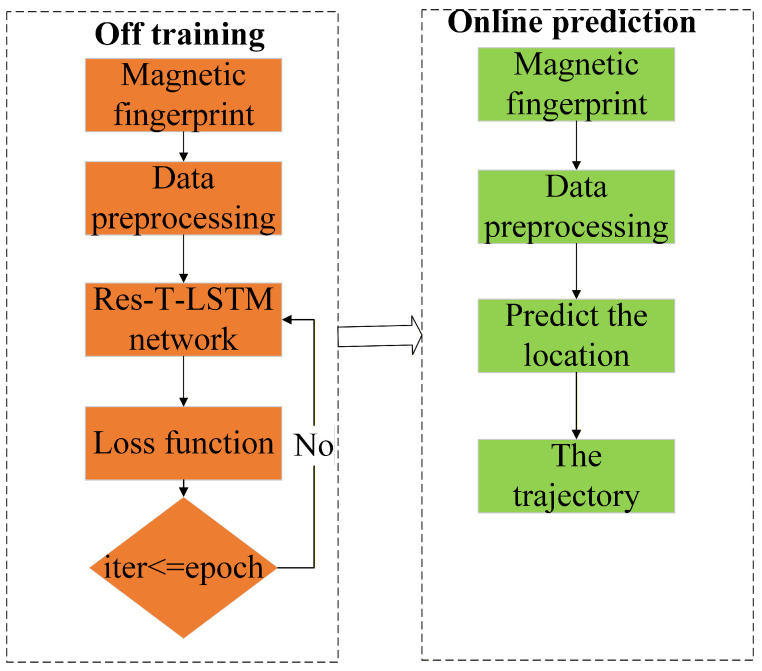
Flowchart of the magnetic fingerprint positioning algorithm using Res-T-LSTM.

Figure 1 shows the overall flowchart of the magnetic-fingerprint positioning algorithm using the Res-T-LSTM model. The framework consists of two main stages: offline training and online prediction. In the offline stage, magnetic-fingerprint data collected from the indoor environment is preprocessed to normalize feature distributions. The processed data is then fed into the neural network for iterative training. The loss function continuously evaluates the discrepancy between predicted and ground-truth positions. If the iteration count has not reached the predefined number of epochs, model parameters are updated and training continues; otherwise, the final positioning model is obtained. In the online stage, real-time magnetic-fingerprint measurements undergo the same preprocessing and are then input into the trained Res-T-LSTM model to infer the current indoor location. Finally, the predicted positions are sequentially combined to generate a continuous user trajectory.

### 3.1. Offline Training

#### 3.1.1. Data Preprocessing

**Figure 2:**
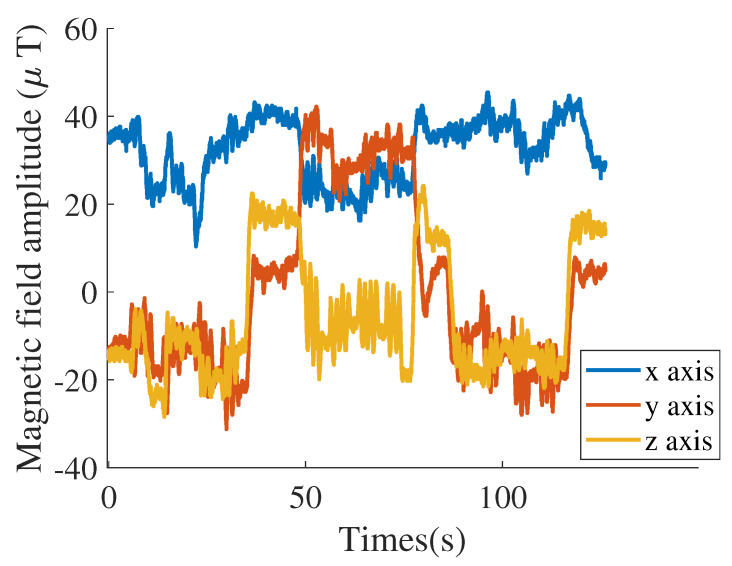
The triaxial magnetic field.

The smartphone acquires triaxial magnetic field data through built-in sensors, denoted as $mag_{x}$, $mag_{y}$, and $mag_{z}$ (Figure 2). To improve stability and reliability during training, the magnetic fingerprint is introduced as a key feature. Calculating the magnetic fingerprint reduces fluctuations caused by changes in magnetic field orientation, allowing the network to capture intrinsic magnetic characteristics and improving recognition accuracy and generalization. The magnetic fingerprint $mag_{F}$ is computed as follows:

(1) $$mag_{F}=\sqrt{mag_{x}^{2}+mag_{y}^{2}+mag_{z}^{2}}$$

Given that natural magnetic field intensities typically remain below 65 μT, the data were normalized to reduce fluctuations during training and enhance model performance. This normalization scales magnetic field intensity values to a range suitable for model training. The normalization procedure is defined as follows:

(2) $$mag_{nor}=mag_{F}/65$$

where $mag_{nor}$ represents the normalized magnetic field.

#### 3.1.2. Res-T-LSTM Network

**Figure 3:**
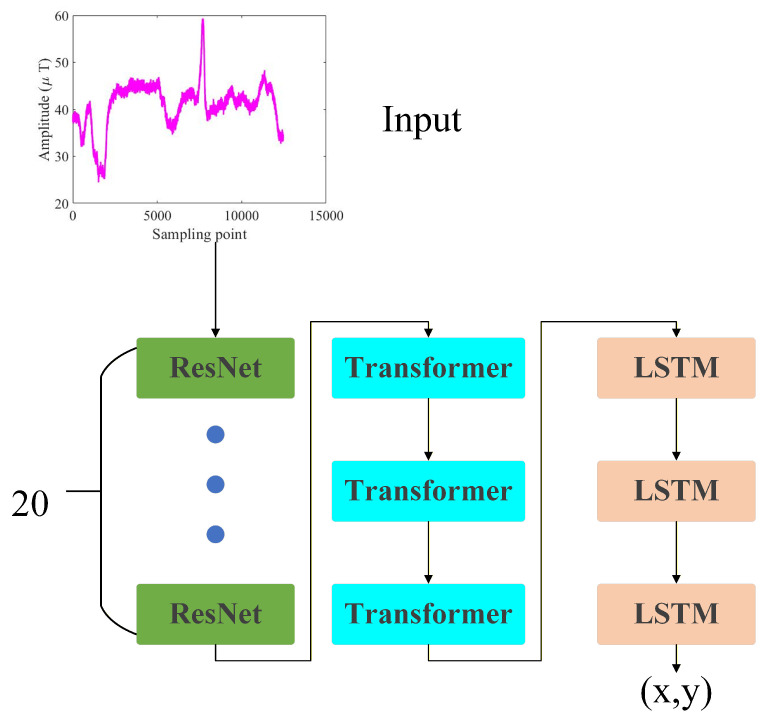
Magnetic fingerprint training network framework.

To fully exploit the rich spatiotemporal features in magnetic sequences collected by smartphones, a novel hybrid network is proposed, as shown in Figure 3. The model comprises 20 ResNets, 3 transformer modules, and 3 LSTM units, enabling simultaneous capture of the temporal evolution of magnetic signals and their local spatial patterns, thereby addressing the limitations of conventional methods in feature extraction and positioning accuracy.

**Figure 4:**
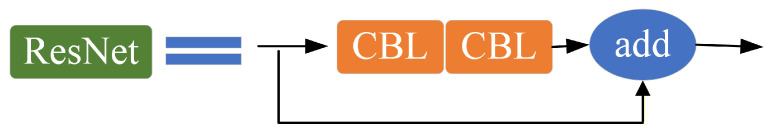
Residual network (ResNet) architecture.

**Figure 5:**
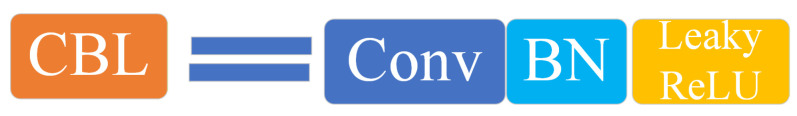
Custom convolutional modules.

The network receives magnetic field magnitude sequences as input, with each sequence comprising multiple sampled intensity values. The input is first processed by a multi-layer ResNet to extract features. Each residual block comprises two custom convolutional modules and a residual connection (Figure 4). Each custom convolutional module contains Conv2D, batch normalization, and an activation function, with its structure detailed in Figure 5. This design effectively captures local discriminative features and enhances noise robustness. To leverage spatial variability in magnetic fingerprints, the network uses 20 residual blocks, enhancing feature representation and maintaining stability in complex environments. Residual connections also mitigate the vanishing gradient problem, enabling efficient deep network training and richer spatial pattern learning. This multi-layer feature extraction mechanism maintains robustness and significantly enhances sensitivity to subtle magnetic variations.

**Figure 6:**
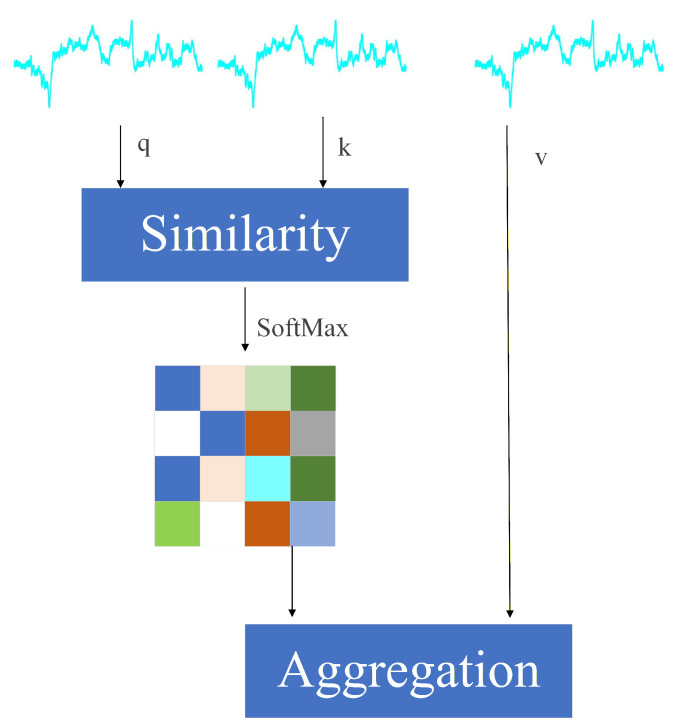
Attention mechanism calculation process.

The extracted magnetic fingerprint feature sequences are processed by an encoder composed of multiple transformer layers. Using a self-attention mechanism, the encoder captures long-range dependencies across time steps or spatial positions and models global contextual correlations, thereby generating comprehensive and discriminative feature representations (Figure 6). Compared with traditional convolutional or recurrent neural networks, the transformer offers stronger global perception and parallel computing capabilities when processing long sequences, enabling full exploitation of spatiotemporal patterns and underlying characteristics in magnetic fingerprint signals. This representation preserves local variation information and integrates global sequence attributes, ultimately improving positioning accuracy and stability in complex environments and dynamic device conditions.

**Figure 7:**
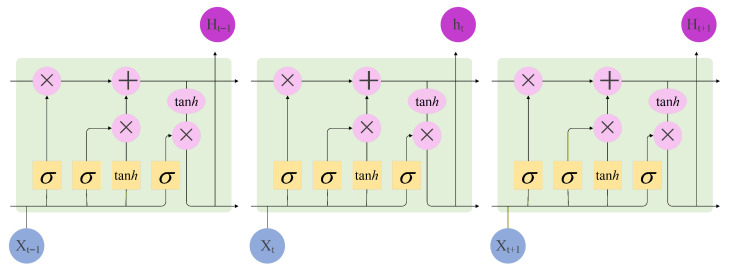
LSTM calculation process.

The extracted magnetic features are subsequently fed into an LSTM network for deep temporal modeling, as illustrated in Figure 7. Through its gating mechanisms, the LSTM effectively preserves historical information while filtering redundant noise, thereby fully leveraging the continuity and evolutionary patterns of magnetic signals. This design further strengthens the intrinsic correlations among spatiotemporal features and enables precise characterization of dynamic trajectories. Finally, the temporally fused high-dimensional features are passed into a regression layer to predict the target’s coordinates (x,y) on the two-dimensional plane.

#### 3.1.3. Loss Function

The loss function quantifies the discrepancy between the model’s predictions and ground-truth positions, serving as a key metric to guide parameter updates and optimize performance during training. In localization regression tasks, the mean squared error is among the most commonly used loss functions. the mean squared error computes the average squared differences between predicted coordinates and true locations, imposing larger penalties on greater deviations and encouraging the model to progressively reduce positioning errors while improving prediction stability and accuracy. Its formulation is defined as follows:

(3) $$Loss=\frac{1}{N}\sum_{i=1}^{N}{\left({\left[\begin{array}{c} x\\ y \end{array}\right]}_{est}-{\left[\begin{array}{c} x\\ y \end{array}\right]}_{true}\right)}^{2}$$

where ${\left[\begin{array}{c} x\\ y \end{array}\right]}_{est}$ and ${\left[\begin{array}{c} x\\ y \end{array}\right]}_{true}$ denote the predicted coordinates and the corresponding ground-truth locations, respectively, and *N* represents the total number of points.

### 3.2. Online Prediction

During prediction, triaxial magnetic field data is collected from the target area and normalized to reduce the influence of magnitude variations on model predictions. The preprocessed data is then input to the positioning model trained offline. The model extracts and analyzes magnetic fingerprint features, leverages spatiotemporal correlations to infer the device’s precise location, and outputs predicted coordinates on a two-dimensional plane, enabling real-time positioning. This process ensures the model adapts to dynamic environments while maintaining high localization accuracy.

## 4. Experiments

### 4.1. Experimental Setup

Experiments were conducted in a 20 m × 20 m × 3.5 m university laboratory, furnished with benches, desktop computers, chairs, square columns, air conditioners, and windows. Participants collected magnetic field data using a Nexus 5X smartphone, with the device held in four postures: calling, dangling, handheld, and pocketed. The smartphone specifications are summarized in Table 1. Participant walking speeds ranged from 0.8 to 1.2 m/s, and the smartphone sampled data at 50 Hz.

The algorithm is trained using the Adam optimizer, with an initial learning rate set to $10^{-4}$ that gradually decays to $10^{-5}$. A warm-up phase is applied to the learning rate, followed by further optimization via cosine annealing. Experiments are implemented in TensorFlow and executed on an NVIDIA RTX 3090 Ti GPU. The high-performance GPU significantly accelerates training, enabling extensive iterations within a reasonable timeframe. This hardware configuration ensures efficient training and supports the model’s strong performance in location prediction tasks.

To comprehensively evaluate the performance of the improved algorithm, four metrics are employed: average error (AE), root mean square error (RMSE), maximum error, and circular error probability (CEP). These metrics are defined as follows, providing a quantitative basis for evaluating the accuracy, consistency, and reliability of the proposed method.

(4) $$e\left(k\right)=\sqrt{{\left(xx\left(k\right)-\hat{xx}\left(k\right)\right)}^{2}+{\left(yy\left(k\right)-\hat{yy}\left(k\right)\right)}^{2}}$$

(5) $$e_{AE}=\frac{1}{N}\Sigma_{k=1}^{k=N}e\left(k\right)$$

(6) $$e_{RMSE}=\sqrt{\frac{1}{N}\Sigma_{k=1}^{k=N}e{\left(k\right)}^{2}}$$

(7) $$e_{MAX}=max\left[\begin{array}{cccc} e(1) & e(2) & \cdot \cdot \cdot & e(k) \end{array}\right]$$

(8) $$e_{CEP(75\%)}=min\left\{R:R\geq 0,|e(k):k=1,2,...,N,||e(k)||\leq R|\geq 0.75N\right\}$$

(9) $$e_{CEP(95\%)}=min\left\{R:R\geq 0,|e(k):k=1,2,...,N,||e(k)||\leq R|\geq 0.95N\right\}$$

where *R* denotes the radius of the smallest circle enclosing the error points. These metrics provide a comprehensive evaluation of the algorithm’s accuracy and reliability. Detailed definitions and calculation procedures can be found in [34].

### 4.2. Experimental Results

#### 4.2.1. Training Loss

**Figure 8:**
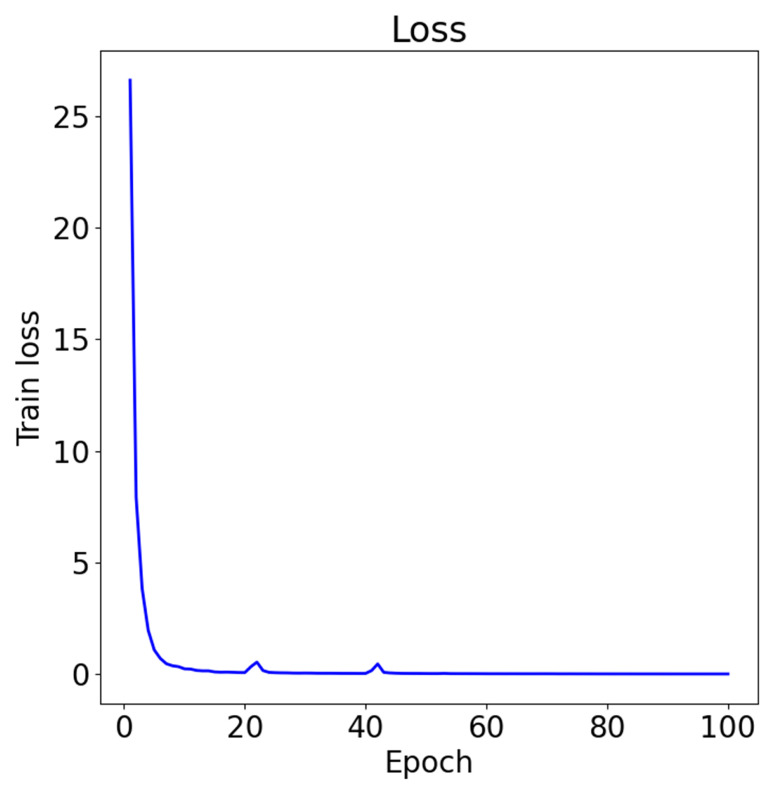
Train losses.

Figure 8 illustrates the evolution of training loss across the entire training process. Overall, the loss decreases steadily with increasing epochs, indicating stable learning and effective convergence of the magnetic-fingerprinting model. In the early stage (approximately the first 3–5 epochs), the loss drops sharply from about 27 to nearly 1, suggesting that the model rapidly captures the fundamental magnetic-fingerprint features. As training progresses, the rate of decrease slows, and minor fluctuations appear around epochs 20 and 40; however, these variations are small and do not disrupt the overall downward trend. After roughly 40 epochs, the loss curve enters a stable region and gradually approaches zero, indicating that the model has essentially converged. These observations indicate that the training process is stable, well-behaved, and free from oscillation or divergence.

#### 4.2.2. Pedestrian Trajectory in the Laboratory

**Figure 9:**
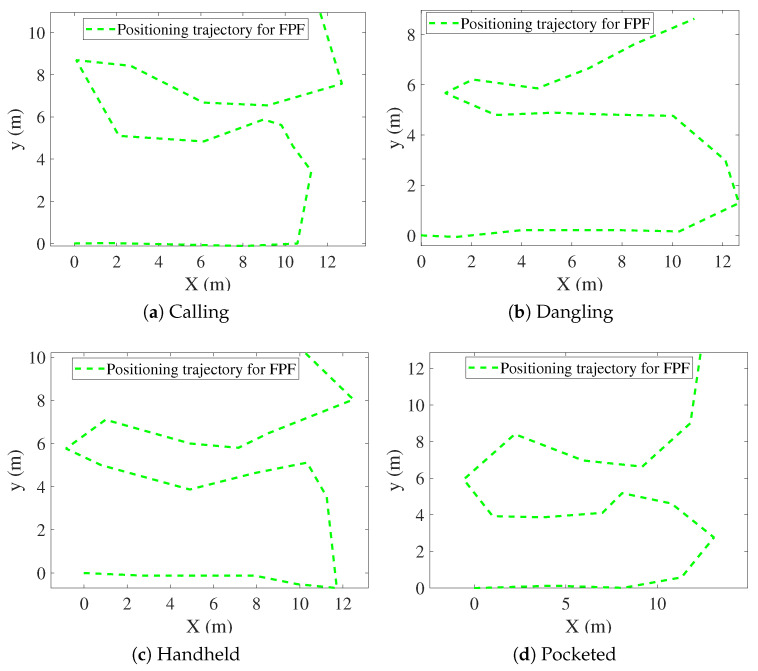
Positioning trajectories with different modes for FPF.

**Figure 10:**
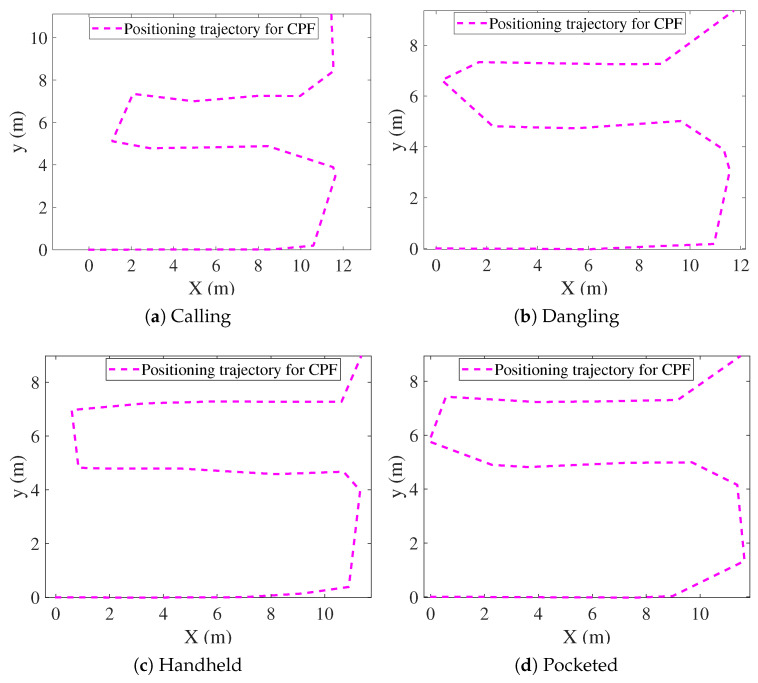
Positioning trajectories with different modes for CPF.

**Figure 11:**
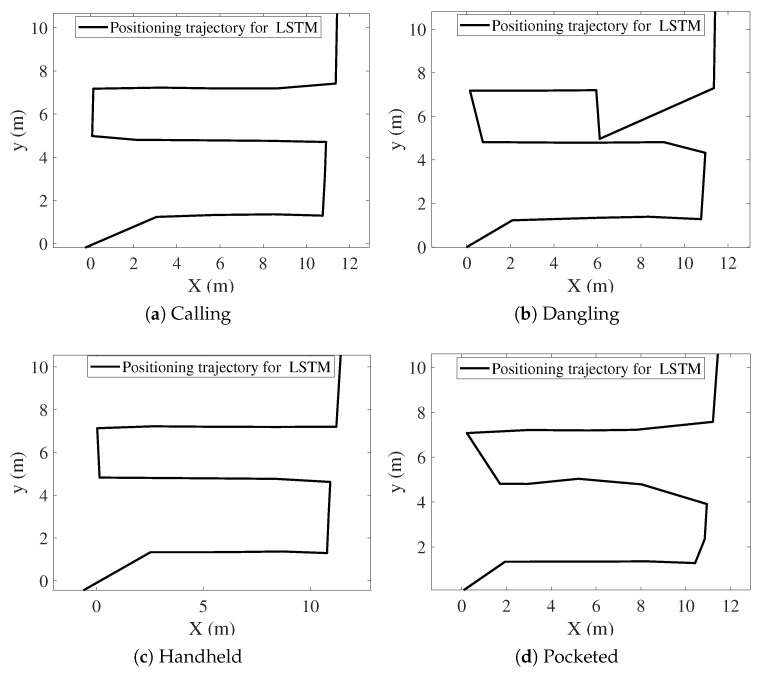
Positioning trajectories with different modes for LSTM.

**Figure 12:**
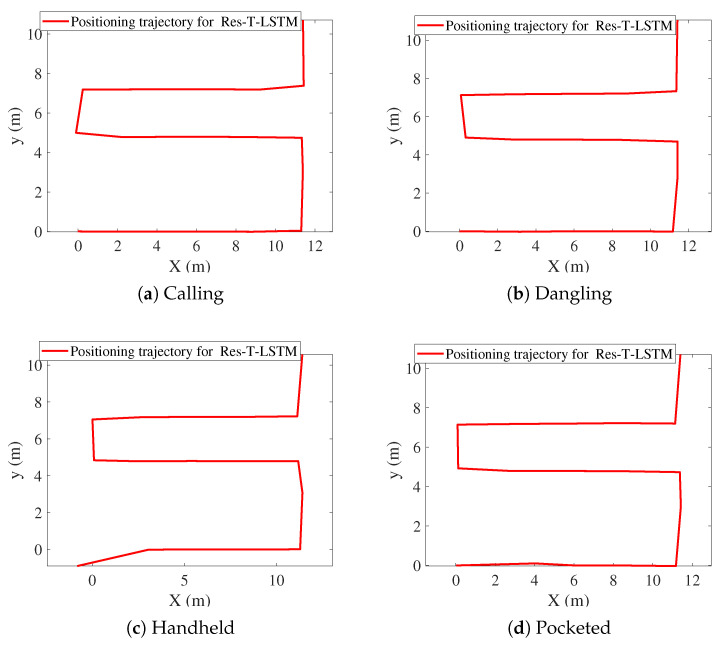
Positioning trajectories with different modes for Res-T-LSTM.

**Figure 13:**
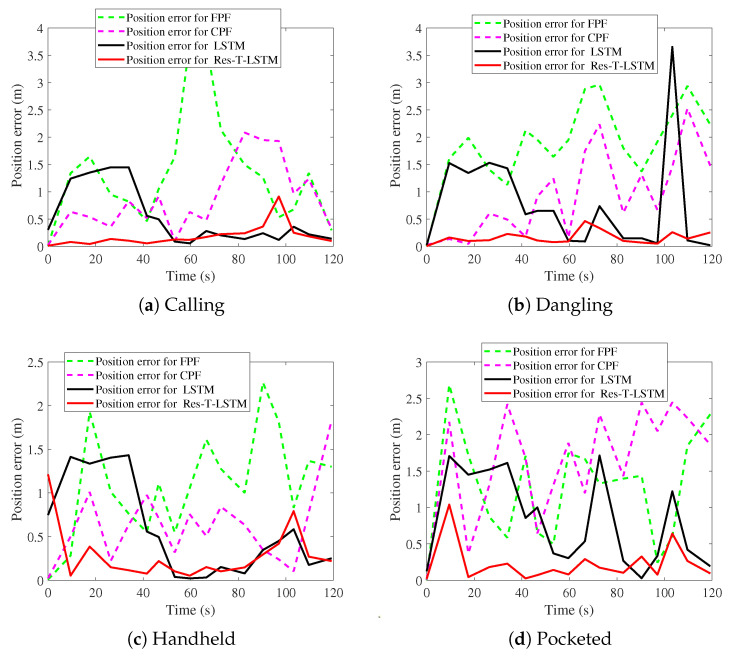
Position errors with different modes.

**Figure 14:**
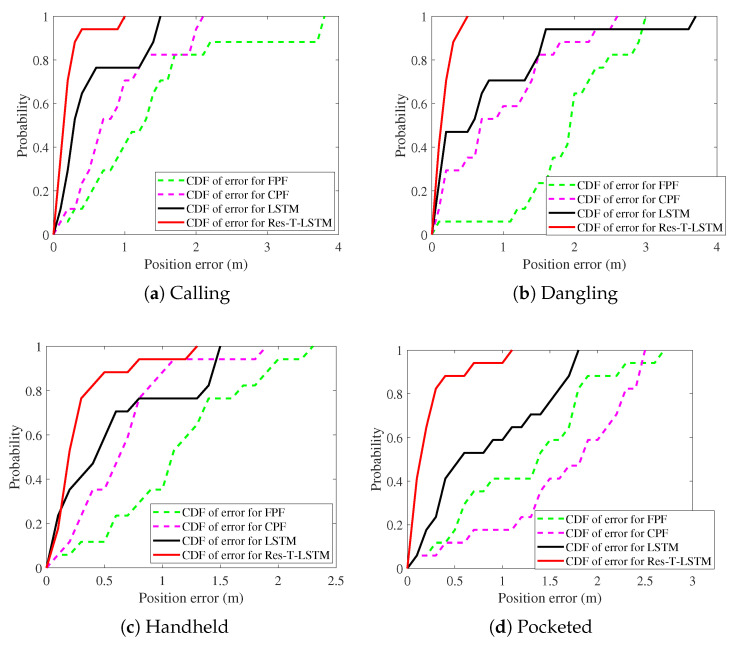
CDFs of errors with different modes.

Figure 9, Figure 10, Figure 11, Figure 12, Figure 13 and Figure 14 present the positioning trajectories, positioning errors, and cumulative distribution functions (CDFs) for the FPF [35], CPF [36], LSTM [37], and Res-T-LSTM under four smartphone placement modes: calling, dangling, handheld, and pocketed. As shown in the figures, the FPF, CPF, and LSTM methods exhibit noticeable deviations and distortions in certain regions, particularly at turning points and trajectory segments with large dynamic posture changes, leading to pronounced positioning errors. These errors mainly arise from reduced magnetic-fingerprint matching accuracy or instabilities in inertial features. In contrast, the proposed Res-T-LSTM method maintains superior accuracy in preserving the overall trajectory shape, capturing key turning points, and ensuring trajectory continuity, effectively suppressing cumulative error propagation.

Table 2 summarizes the positioning errors of the FPF, CPF, LSTM, and the proposed Res-T-LSTM models under four smartphone placement modes. Several observations can be drawn.

FPF and CPF exhibit relatively large positioning errors, particularly in dynamic scenarios such as the dangling and pocketed modes, where rapid device-orientation changes weaken the reliability of magnetic fingerprint matching. For instance, FPF yields an RMSE of 2.03 m in the dangling mode, while CPF reaches an RMSE of 1.79 m in the pocketed mode, indicating noticeable performance degradation under unstable motion conditions.

The LSTM-based method improves overall positioning accuracy compared with fingerprint-based approaches, especially in handheld and calling modes where motion remains relatively stable. However, its performance still declines in highly dynamic user behaviors. For example, in the dangling mode, LSTM exhibits an RMSE of 1.18 m, primarily due to cumulative drift caused by unstable inertial estimates.

In contrast, the proposed Res-T-LSTM consistently achieves the lowest positioning errors across all scenarios. By integrating spatial feature extraction with temporal dependency modeling, it effectively suppresses cumulative drift and enhances robustness to abrupt changes in device posture. Notably, the RMSE values in the dangling and pocketed modes are reduced to only 0.2 m and 0.34 m, respectively, representing substantial performance improvements over competing methods.

The proposed model achieves an RMSE of 0.3 m across the four placement modes, significantly outperforming CPF (1.19 m), FPF (1.6 m), and LSTM (0.91 m). These results confirm the superior accuracy, stability, and generalization capability of the Res-T-LSTM approach for indoor magnetic positioning.

#### 4.2.3. Ablation Study

Table 3 presents the ablation study results comparing the LSTM, T-LSTM, and the proposed Res-T-LSTM models under four smartphone placement modes. Several noteworthy findings can be summarized.

The baseline LSTM model produces the largest positioning errors across all placement modes, primarily due to its limited ability to capture long-range temporal dependencies and insufficient modeling of spatial feature variations. In the pocketed and dangling modes, the RMSE reaches 10.19 m and 12 m, respectively, indicating significant cumulative drift under strong motion disturbances.

With the introduction of the transformer module, the T-LSTM model considerably improves positioning accuracy, reducing the RMSE to 1.56 m and 1.39 m in the pocketed and dangling modes. This result verifies the contribution of enhanced temporal feature representation in suppressing drift and improving robustness to dynamic motion patterns.

The proposed Res-T-LSTM achieves the best performance across all scenarios. Through spatial feature extraction and residual temporal modeling, the RMSE is further reduced to 1.04 m and 0.46 m in the pocketed and dangling modes, corresponding to reductions of 89.8% and 96.2% relative to the baseline LSTM. On average, the Res-T-LSTM attains an RMSE of 0.91 m, outperforming both T-LSTM (1.11 m) and LSTM (9 m).

Overall, the ablation results indicate that both the transformer module and residual spatial representation contribute significantly to performance improvement, and their integration plays a key role in achieving accurate and robust indoor magnetic positioning.

## 5. Discussions

Experimental results demonstrate that the Res-T-LSTM framework substantially enhances magnetic-fingerprint positioning performance in complex indoor environments. This section explores the underlying mechanisms behind its performance improvement, the model’s limitations, and the practical implications of the findings.

The success of Res-T-LSTM primarily arises from the synergistic modeling capability of its hybrid architecture in capturing the spatiotemporal characteristics of magnetic signals. Compared with traditional methods that rely on local sequence matching, the transformer module captures global contextual dependencies within magnetic sequences via self-attention mechanisms. This enables the model to not only compare local segments but also interpret the significance of each measurement within the context of the entire trajectory, thereby providing greater robustness against sequence stretching or compression due to varying walking speeds. For instance, under highly dynamic modes, traditional methods experience significant error increases due to magnetic signal distortion, whereas Res-T-LSTM maintains stable accuracy.

Furthermore, deep spatial features extracted by the ResNet module enhance the sensitivity to subtle magnetic fluctuations. Metal structures generate unique local magnetic features. Residual connections in ResNet effectively mitigate gradient vanishing, ensuring that these critical features are preserved throughout deep networks. LSTM then models the temporal evolution of these spatial features, effectively mitigating drift caused by cumulative errors in inertial sensor estimations.

Despite its excellent performance, this study has several limitations. First, experiments were confined to a laboratory, where magnetic patterns are likely less complex than those in large public venues, which exhibit stronger multipath interference and dynamic pedestrian flows. Second, data were collected using a single smartphone model, without accounting for magnetometer heterogeneity across devices, which may affect positioning consistency in real-world cross-device deployments. Finally, relying solely on geomagnetic data renders the system potentially vulnerable to sudden magnetic disturbances.

## 6. Conclusions

This paper proposes a hybrid deep learning framework, Res-T-LSTM, to tackle challenges arising from magnetic fingerprint sequence distortion and instability under varying walking speeds, device orientations, and dynamic indoor environments. The framework integrates ResNet for deep spatial feature extraction, transformer for modeling global contextual dependencies, and LSTM for temporal dynamics learning, effectively capturing the complex spatiotemporal characteristics of magnetic signals.

Experimental evaluations show that Res-T-LSTM consistently outperforms traditional magnetic fingerprinting methods and single-module deep learning models across four smartphone-carrying modes, achieving an average positioning error of 0.21 m in a 20 m × 20 m laboratory. Ablation studies further confirm the critical and complementary contributions of the transformer’s global modeling capability and ResNet’s spatial feature extraction.

Future work will focus on three directions. First, validating the method in larger, structurally complex real-world indoor environments to assess its generalization capability. Second, investigating domain adaptation and cross-device calibration techniques to mitigate the impact of sensor heterogeneity. Finally, exploring the fusion of geomagnetic data with multi-modal sensing to enhance system robustness across diverse conditions. 

## Figures and Tables

**Table 1:** Technical specifications of the smartphone.

Nexus 5X	CPU	GPU	RAM	OS
	Qualcomm Snapdragon 808	Adreno 418	2 GB LPDDR3	Android 6.0

**Table 2:** Position errors for FPF, CPF, and Res-T-LSTM (m).

Motion Modes	Error	AE	RMSE	ME	CEP of 75%	CEP of 95%
Calling mode	FPF	1.36	1.7	3.74	1.64	3.76
	CPF	0.87	1.06	2.08	1.18	2.02
	LSTM	0.51	0.71	1.45	0.58	1.46
	Res-T-LSTM	0.19	0.27	0.91	0.23	0.92
Dangling mode	FPF	1.9	2.03	2.96	2.28	2.96
	CPF	0.93	1.2	2.53	1.44	2.52
	LSTM	0.75	1.18	3.66	1.38	3.62
	Res-T-LSTM	0.16	0.2	0.46	0.23	0.42
Handheld mode	FPF	1.1	1.24	2.27	1.39	2.22
	CPF	0.61	0.74	1.83	0.79	1.82
	LSTM	0.56	0.76	1.43	0.78	1.47
	Res-T-LSTM	0.28	0.41	1.21	0.29	1.22
Pocketed mode	FPF	1.25	1.45	2.69	1.76	2.62
	CPF	1.63	1.79	2.45	2.24	2.47
	LSTM	0.8	1	1.71	1.48	1.76
	Res-T-LSTM	0.22	0.34	1.04	0.25	1.01
General	FPF	1.4	1.6	2.91	1.76	2.89
	CPF	1.01	1.19	2.22	1.41	2.2
	LSTM	0.66	0.91	2.06	1.05	2.08
	Res-T-LSTM	0.21	0.3	0.91	0.25	0.89

**Table 3:** Position errors for LSTM, T-LSTM, and Res-T-LSTM (m).

Motion Modes	Error	AE	RMSE	ME	CEP of 75%	CEP of 95%
Calling mode	LSTM	3.66	4.18	7.88	4.18	7.82
	T-LSTM	0.32	0.36	0.62	0.43	0.62
	Res-T-LSTM	0.19	0.27	0.91	0.23	0.92
Dangling mode	LSTM	4.22	5.12	12	5.38	11.92
	T-LSTM	0.43	0.58	1.39	0.66	1.32
	Res-T-LSTM	0.16	0.2	0.46	0.23	0.42
Handheld mode	LSTM	2.89	3.31	5.93	3.84	5.92
	T-LSTM	0.43	0.51	0.88	0.64	0.87
	Res-T-LSTM	0.28	0.41	1.21	0.29	1.22
Pocketed mode	LSTM	3.66	4.55	10.19	4.28	10.12
	T-LSTM	0.59	0.74	1.56	0.78	0.1.56
	Res-T-LSTM	0.22	0.34	1.04	0.25	1.01
General	LSTM	3.61	4.29	9	4.42	8.94
	T-LSTM	0.44	0.55	1.11	0.62	1.09
	Res-T-LSTM	0.21	0.3	0.91	0.25	0.89

## Data Availability

Data presented in this study are available on request from the corresponding author.

## Abbreviations

The following abbreviations are used in this manuscript:

ResNet	Residual networks
PDR	Pedestrian dead reckoning
CNN	Convolutional neural network
RNN	Recurrent neural network
CDFs	Cumulative distribution functions
AE	Average error
RMSE	Root mean square error
ME	Maximum error
CEP	Circular error probability
